# Spatiotemporal sentiment variation analysis of geotagged COVID-19 tweets from India using a hybrid deep learning model

**DOI:** 10.1038/s41598-022-05974-6

**Published:** 2022-02-03

**Authors:** Vaibhav Kumar

**Affiliations:** Data Science and Engineering, Indian Institute of Science Education and Research, Bhopal, 462066 India

**Keywords:** Sustainability, Computer science, Information technology

## Abstract

India is a hotspot of the COVID-19 crisis. During the first wave, several lockdowns (L) and gradual unlock (UL) phases were implemented by the government of India (GOI) to curb the virus spread. These phases witnessed many challenges and various day-to-day developments such as virus spread and resource management. Twitter, a social media platform, was extensively used by citizens to react to these events and related topics that varied temporally and geographically. Analyzing these variations can be a potent tool for informed decision-making. This paper attempts to capture these spatiotemporal variations of citizen reactions by predicting and analyzing the sentiments of geotagged tweets during L and UL phases. Various sentiment analysis based studies on the related subject have been done; however, its integration with location intelligence for decision making remains a research gap. The sentiments were predicted through a proposed hybrid Deep Learning (DL) model which leverages the strengths of BiLSTM and CNN model classes. The model was trained on a freely available Sentiment140 dataset and was tested over manually annotated COVID-19 related tweets from India. The model classified the tweets with high accuracy of around 90%, and analysis of geotagged tweets during L and UL phases reveal significant geographical variations. The findings as a decision support system can aid in analyzing citizen reactions toward the resources and events during an ongoing pandemic. The system can have various applications such as resource planning, crowd management, policy formulation, vaccination, prompt response, etc.

## Introduction

The world is witnessing a global pandemic of COVID-19^1^. India is at the centre of this pandemic. Around 10.7 million confirmed cases and around two lakh deaths in 28 states and eight union territories of India were reported from the first COVID-19 case on January 30 2020, till the end of the first wave which roughly lasted till October 2020^2^. To break the chain of spread Government of India (GOI) announced 21 days nationwide lockdown starting from March 25, 2020^3^. States were made more accountable to set up plans for stopping the spread of the virus^4^. The lockdown was extended in three more phases, and after that four gradual unlock phases were applied with each phase having some ease and restrictions on the activities. Table 1 details the corresponding activity permissions during the lockdown and unlock phases.

**Table 1:** COVID-19 lockdown and unlock in India, and allowed and restricted activities (based on notifications issued by (GOI).

Phase	Acronym	Period	Restricted and allowed activities
Lockdown 1	L1	March 25–April 14	Restriction on all outdoor activities except essential services
Lockdown 2	L2	April 15–May 3	Restriction on all outdoor activities except essential services
Lockdown 3	L3	May 4–May 17	Restriction on all outdoor activities except essential services, agricultural, construction, and few industrial activities
Lockdown 4	L4	May 18–May 31	Movement of goods cargo (including rickshaws and auto-rickshaws, empty cargo vehicles, taxis and cab aggregators. Interstate movement of passenger vehicles/buses) and hospitality services were allowed
Unlock 1	U1	June 1–June 30	Interstate movement of vehicles allowed, special trains on selected routes, domestic air travel, all commercial and industrial activities with time restrictions. Hospitality services were allowed with half capacity, night curfew from 9 PM till 5 AM
Unlock 2	U2	July 1–July 31	Same as unlock 1, more trains and domestic flights allowed, industrial units in multiple shifts were allowed, night curfew from 10 PM to 5 AM
Unlock 3	U3	August 1–August 31	Same as unlock 2. All recreational/ cultural/ social/ political/ academic/ religious/ entertainment functions and other large gatherings were not allowed
Unlock4	U4	September 1–September 30	In areas outside containment zones all activities were allowed except schools and colleges remained closed, and operation of special trains was increased

India with a staggering population of more than 1.3 billion is a resource-constrained country with many complex socioeconomic systems that vary along the vast geographic regions. The diversity also influenced day-to-day activities during COVID-19 pandemic in unique ways. This made efficient resource planning even more challenging; as a result, the population becomes more vulnerable to the virus spread and poor service delivery. Citizens across the country shared their views and reactions to the events that unfolded during lockdown and unlock phases^4^ over a widely known social media platform “Twitter”. For example, during the massive reverse migration of daily wagers triggered fear of virus spread and the event drew sharp reactions. These reactions were mainly related to mental state, virus, and resources such as daily requirements, emergency equipment, health, food, and travel that varied across the country. Analysis of sentiments using contextual mining of the tweets helps understand the subjective social sentiment on many topics^5,6^. Hence, it can be an effective medium to analyze reactions to COVID-19 related developments^7^. It can also help capture the mood of the nation, which is often a driving force in decision making^8^. Moreover, integrating the sentiments with location information using Geographic Information Systems (GIS) can be further enhanced using analysis such as hotspots and clusters identification. Such analysis can help the agencies develop evidence driven policy instruments for efficient infrastructure planning during pandemic ^7^.

Over the years, Twitter has been used in many decision-making systems (e.g., ^9–13^). Twitter and its applications in COVID-19 issues have also been studied by various researchers^14–16^. Sentiment analysis from Twitter data is considered a potent tool in quantifying reactions to an event. The reader can refer to a review by Alamoodi et al.^17^ concerning sentiment analysis and its applications in fighting COVID-19 and past infectious diseases for more details. In the context of COVID-19, limited studies have captured the citizens' sentiment for a specific country^18,19^. Moreover, the classification and analysis of sentiments during lockdown and unlock phases in India still remains a gap. The paper aims to address this gap.

With the advancements in Machine Learning (ML), specifically Deep Learning (DL), the ability to analyse text is in an exhilarating phase^20^. Many recent studies (e.g., ^13,17,21–27^) have implemented Deep Learning (DL) models for contextual mining of COVID-19 data. However, the model classes, i.e., CNN and RNN, applied in these studies suffer from various limitations. For example, RNN class models often fail to capture different local associations between parts of long texts. On the other hand CNN class of models does not consider the long-term dependencies between word sequences, which are essential in natural-language processing (NLP) tasks^28^. Moreover, selecting suitable filter size for text classification in CNN remains a challenge^28,29^. Research in the recent few years by integrating the two model classes (RNN variants and CNN) has successfully utilized CNN's property for extracting features and LSTM to support sequence-based prediction. Due to their complementing, their integrated architectures perform significantly better than when implemented individually. For example, CNN + BiLSTM by Yenter & Verma^29^ takes advantage of CNN property to learn the spatial structure and combines it with LSTM for sentiment analysis. X. Wang et al.^30^ combined CNN and RNN to extract local features using CNN and long-term dependencies using RNN. J. Wang et al.^31^ integrated CNN-LSTM for Dimensional sentiment analysis.

Motivated by the existing research, this study aims to leverage the strengths of BiLSTM and CNN model classes to perform contextual mining and predict the sentiment of COVID-19 tweets, which has not been attempted yet. Further, despite being the hotspot of the discussion around COVID-19, to the best of my knowledge no study regarding sentiment analysis has been done for India during the lockdown and unlock phases.

Moreover, no study has analyzed the spatiotemporal sentiment patterns of the geotagged tweets for India during the lockdown and unlock phases. The primary objective of the paper is to fill this gap. The methodology and the outcomes presented in the paper are targeted to support the policymakers as a decision support system. The system while analysing the citizen sentiments with high accuracy, attained using the proposed deep learning model can aid in formulating policies, plan of actions, resource planning, emergency management, etc., to fight the pandemics like COVID-19. Further, one of the paper's objectives is to propose an information transfer framework to support the decision support system. The major contributions of the paper are as follows:

- Development of a hybrid deep learning model by integrating a BiLSTM followed by a CNN model for sentiment classification of the geotagged novel COVID-19 tweets from India done during the lockdown and unlock phases.
- Comparison of model outcomes with various widely discussed machine learning architectures, i.e., CNN, BiLSTM, CNN + BiLSTM (CNN followed by BiLSTM)
- Spatiotemporal pattern analysis of the predicted sentiments using GIS.
- A discussion on the proposed conceptual framework to support the system through an information transfer process during pandemics such as COVID-19.

The paper is organized as follows. Methodology is discussed in the next section. The model architecture is discussed in the “Proposed Model Architecture” section. The results are presented in the “Results” section. Discussion and policy recommendations are elucidated in the “Discussion and Utility of the Research” section, followed by conclusion in the last section.

## Methodology

**Figure 1:**
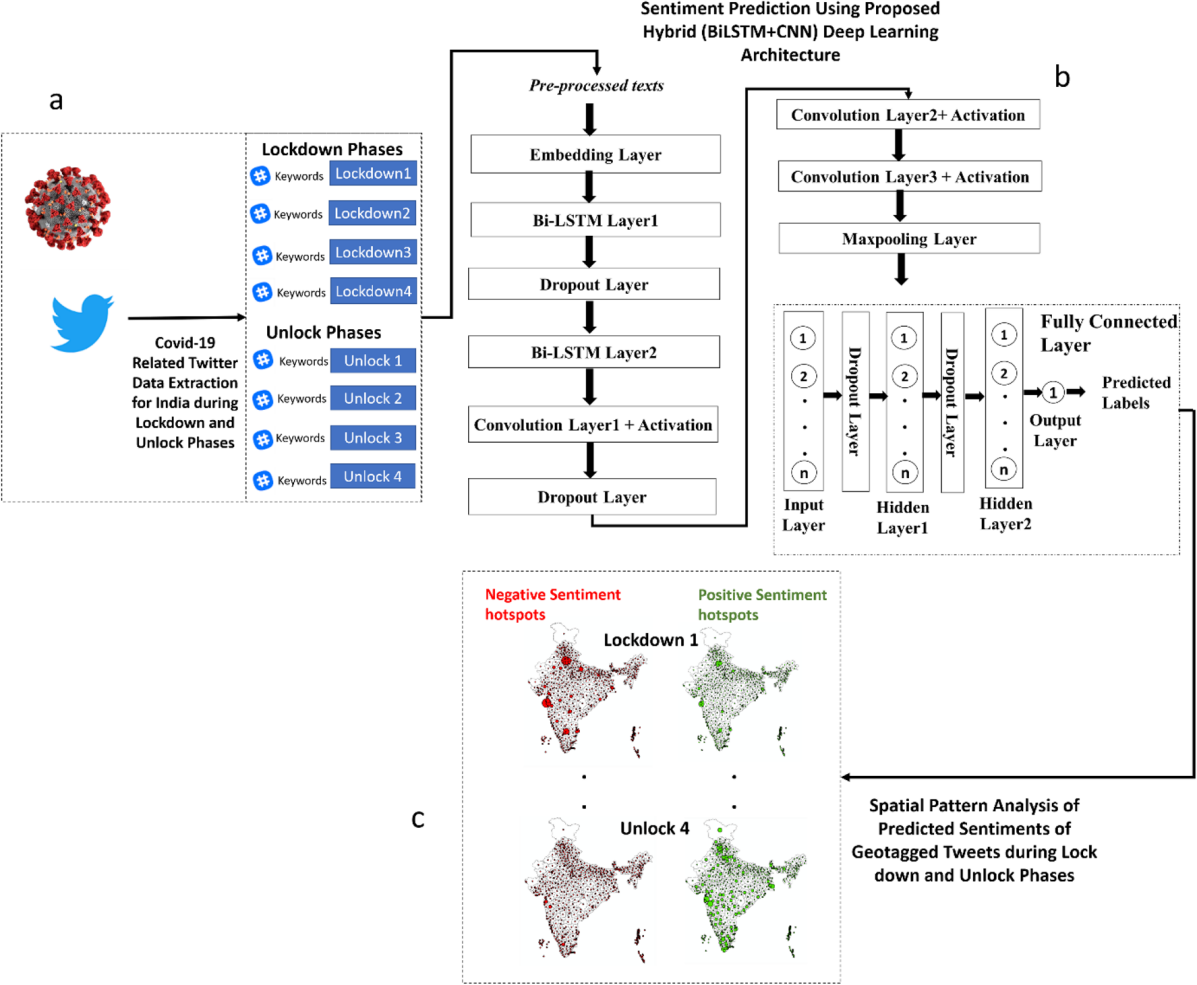
Methodological framework (**a**) data collection and preprocessing (**b**) model development (**c**) spatiotemporal analysis of predicted sentiment of geotagged tweets.

Figure 1 illustrates the methodological framework of the research in three major steps. These steps are discussed in detail in the subsections that follow.

## Dataset preparation and preprocessing

### Data collection

The real-time Twitter feed was accessed through the streaming API of Twitter^32^ from March 23, 2020 onwards for India. Besides, the data published by Twitter on May 13, 2020, which was prepared using various keywords and location bounding boxes was also used. The keywords were emerging continuously throughout the study, primarily due to changing events during lockdown and unlock phases. Hence, the keywords were updated with time by analysing the n-grams after a period. The 'trends’ feature by Twitter was also used to identify the keywords. Table 2 illustrates the overview of keywords used to extract the data. This study only focuses on the tweets done in the English language. This can also lead to some information loss as India has several languages. Thus, consideration of multilingual tweets remains a limitation of the study.

**Table 2:** Overview of the keywords used to collect data during lockdown and unlock phases.

Period	Keywords
March 23–30 April 2020	corona, covid19, coronavirus, chinavirus, quarantine, safety, covidcase, lockdown, sarscov2, ncov2019, pandemic, wearamask, socialdistancing, stayathome, stayhome, migrants, migrantcrisis, labours
May 1–May 31 2020	**Newly added:** handsanitizer, workfromhome, n95, tablikijamat, ppe, flaatening flattenthecurve, flatteningthecurve, covidwarriors, muslims, goi, Namo, arogyasetu, openbars, frontlineheroes, coronawarriors, asymptotic,
May 1 2020–June 30 2020	**Newly added:** doctorsheros, mumbaicorona coronavaccine, Patanjali, Ramdev , hometasking, wfh, herdimmunity, unlock, virusspread,
June 30 – 30 September	**Newly added:** Washurhands, openbars, shutcollege, opentrains, newnormal, vaccine, vaccinetrials

### Data filtration and geocoding

The collected data were filtered based on the location information. It was done by filtering geotagged tweets for India. A significantly less number of tweets were found geotagged. However, this is reasonable as location sharing in tweets is an option for users. Besides, various studies have discussed similar outcomes that reported that only 0 to 3% of tweets were found geotagged out of the whole tweets^33,34^. A recent study on COVID-19 by Qazi et al.^35^ also reported less than 1% geotagged COVID-19 related tweets out of millions of extracted tweets since February 2020. Table 3 shows the total tweets collected for various phases of lockdown and unlock.

**Table 3:** Total count of the geotagged tweets used as test datasets.

Phase	Total retrieved geotagged tweets
Lockdown 1	19,489
Lockdown 2	14,692
Lockdown 3	14,776
Lockdown 4	14,021
Unlock 1	13,792
Unlock 2	17,728
Unlock 3	16,737
Unlock 4	16,861

### Tweet preprocessing

Tweets were preprocessed to remove extra spaces, special characters, emojis, mentions (@mentions), numbers, URLs, and paragraph breaks. After the initial cleaning all the tweets were tokenized by splitting a string into tokens (individual words) using Keras Tokenizer library. After tokenizing stemming, a rule-based process was applied to strip suffixes (“s”, “ly”, “es”, “ing”, etc.) of the tokens. Advanced preprocessing such as spelling correction, abbreviations to full form conversion were done to avoid the system bottlenecks.

## Proposed model architecture

Many studies have proposed various DL architectures such as Recurrent Neural Network (RNN) and its variant Long Short Term Memory (LSTM) in Text classification^36^. These architectures use sequential patterns and associations between words by treating a sentence as a series of tokens to predict sentiments specific categories such as positive or negative. LSTM can extract text context dependencies better than RNN^10,37^. Still, it faces significant challenges in weighting the word order, i.e., a future text has a more significant impact on the text representation than the preceding one. BiLSTM, a variant of LSTM, has two parallel layers that aim to overcome the capturing context-based limitation of LSTM by operating in both directions, i.e., past to future and future to past. This property makes it suitable for learning long-term associations while negating duplicate information in the text^38^. However, the variants often fail to capture different local associations between parts of long texts, which is tackled by extracting higher-level features using CNN models when combined with sequential^6^.

This study also proposes adding BiLSTM before CNN-based deep learning model (see Fig. 1b) to enhance the learning structure by capturing the sequential relations before feature extraction with less information loss. The input of this network is tweet text as word sequences. The embedding layer transforms the text into word vector of *n* dimension, where *n* is the vocabulary size. Thereafter two sequential BiLSTM models learn the input word vector matrix {*a*_*1*_*, a*_*2*_*, …., a*_*n*_} and yield a layer of the same size {*h*_*1*_*, h*_*2*_*, …., h*_*n*_}. These layers further enhance the embedded semantics. The outcome of the BiLSTM is input to a 1D CNN model which is then sequentially connected to two 1D CNN layers for obtaining local text semantic features. The information of the extracted features were enhanced through maxpooling and then are flattened to serve as inputs to fully connected layers which predict the sentiment of the input text. Regularization through dropout layers was achieved to minimize model overfitting. The dropout functions implemented in the dropout layers penalize large weights to optimize the neural network. Table 4 illustrates the detailed description of the architecture and individual models. The subsequent subsections describe the architectural components in detail.

**Table 4:** The model architecture.

Layer	Properties and dimensions
Embedding Layer (Word Embedding)	Output dimension: 64 Input sequence length: 500
BiLSTM Layer	Forward: Number of hidden nodes: 128 Backward: Number of hidden nodes: 128
Dropout layer	Probability = 0.20
BiLSTM Layer	Forward: Number of hidden nodes: 256 Backward: Number of hidden nodes: 256
Convolution + Activation Layer	Number of filers = 64 Filter size = 5 Activation function: ReLU
Dropout layer	Probability = 0.20
Convolution + Activation Layer	Number of filers = 128 Filter size = 5 Activation function: ReLU
Convolution + Activation Layer	Number of filers = 256 Filter size = 3 Activation function: ReLU
Maxpooling layer	Pool Size: 3 Stride:1
Flatten	
Hidden Layer 1	Number of hidden neurons: 128 Activation function: ReLU
Dropout layer	Probability = 0.15
Hidden Layer 2	Number of hidden neurons: 64 Activation function: ReLU
Output layer	Number of neurons:1 Activation function: Sigmoid

### Sequence embedding layer

A word embedding is a learned depiction of texts, where words with identical meanings have a similar representation. The layer learns the representation of individual input words in a text having a unique identification by initializing with random weights^10,37^. In this study Python Keras library that offers a framework for embedding layer was implemented. The layer requires the input data to be tokenized, which was done using the tokenizer module of Keras. The input dimension argument of the layer, which is the size of the vocabulary was selected as 2000. The output dimension, i.e., size of the vector space in which words will be embedded was defined as 64. Lastly, the input length which corresponds to the length of input sequences was defined as 50 based on the words in the input tweet.

### BiLSTM layers

BiLSTM models in the architecture were used to extend the word embedding outcome of the embedding layer. The features obtained from embedded layer were fed to the first BiLSTM layer and its outcome was input to the second BiLSTM layer. The association between the previous inputs and the output is detected through the sequential order between the data. To avoid overfitting a dropout layer was introduced between the two BiLSTM layers. The main components of the BiLSTM model are the forward (*hl* (*wi*)) and backward (*hr* (*wi*)) output vectors which store the left and right context of a word (*w*_*i*_*)*, calculated using formulas (1) and (2):

1 $$h_{l} = f(W^{l} h_{l} \left( {w_{i - 1} } \right) + W^{el} e\left( {w_{i - 1} } \right) + b_{l}$$

2 $$h_{r} = f(W^{r} h_{r} \left( {w_{i + 1} } \right) + W^{er} e\left( {w_{i + 1} } \right) + b_{r}$$

Here, the word embedding of word *w*_*i*_ is denoted as e (*w*_*i*_). *W*^*l*^ and *W*^*r*^ are forward and backward hidden layer transformation matrixes that change the state of the current state into the next hidden layer in forward and backward directions. *W*^*el*^ and *W*^*er*^ are forward and backward hidden layer transformation matrixes that change the state of the current state into the next hidden layer in forward and backward directions. *W*^*el*^ and *W*^*er*^ weight matrixes transform input word embeddings of forward and backward layers. *b*_*l*_ and *b*_*r*_ are the bias associated with forward and backward layers, respectively. Lastly, *f* is a nonlinear activation function, which in our case is Rectified Linear Unit (ReLU)^39^.

### 1D CNN

In the 1D convolution layers *v* represents input vector and *k* kernel. If the layer *v* has length *m*, and *k* has length *n*, the convolution *v ∗ k* of *v* and *k* is defined as:

3 $$\left( {v*k} \right)\left( j \right) = \mathop \sum \limits_{i = 1}^{i = n} k\left( i \right) v\left( {j - i + \frac{n}{2}} \right)$$

Various convolution kernels with varied widths were convolved over the input vectors to capture the hidden correlations between several adjacent words, i.e., local relationships. In the architecture, the number of kernels in the first second and the last convolution layer were selected as 64, 128, and 256, respectively. The kernels served as n-gram detectors as each kernel assigned high scores to definite class of n-grams. The convolution operation was followed by an activation layer. ReLU, a widely implemented nonlinear activation function was selected for the activation layers. Instead of maxpooling after each convolution, maxpooling operation was implemented after the third convolution layer to combine the vectors resulting from different convolution windows, the largest value of all channel timesteps. The resultant maxpooled vector captures the most relevant features of the sentence. After all convolution-pooling computations were done, the final feature maps were flattened to serve as inputs to fully connected (FC) neural network for sentiment prediction. The FC architecture has two hidden dense layers with 128 followed by 64 neurons. For restraining the model from overfitting a dropout layer was introduced between the hidden layers. The final layer having single neuron applies ‘Sigmoid’ activation function to predict the final outcome, i.e., the binary sentiment label for an input sentence.

To predict the sentiments of the tweets the proposed architecture was trained on a freely available Sentiment140^40^ dataset; the data set contains around 16 lakh tweets and their sentiment labels, i.e., positive and negative. Out of the 16 lakh tweets seventy percent data was used in training and thirty percent data was used for validation. The developed model was tested on manually annotated 20 K tweets on COVID-19. These tweets were from all the lockdown and unlock phases. Twenty-five PhD research scholars from various interdisciplinary backgrounds from Indian Institute of Technology Bombay were involved in the labeling process. Once the testing was done, tweets from various lockdown and unlock phases were predicted for their label using the model. The predicted tweet sentiments were joined using a GIS-based system to generate district-wise sentiments count hotspot maps.

### Ethics declarations

The study was approved by the Ethics committee of the Indian Institute of Science Education and Research Bhopal, India.

### Consent to participate

The need of Informed consent was waived by the Ethics committee of the Indian Institute of Science Education and Research Bhopal, India.

## Results

### Word-cloud analysis

**Figure 2:**
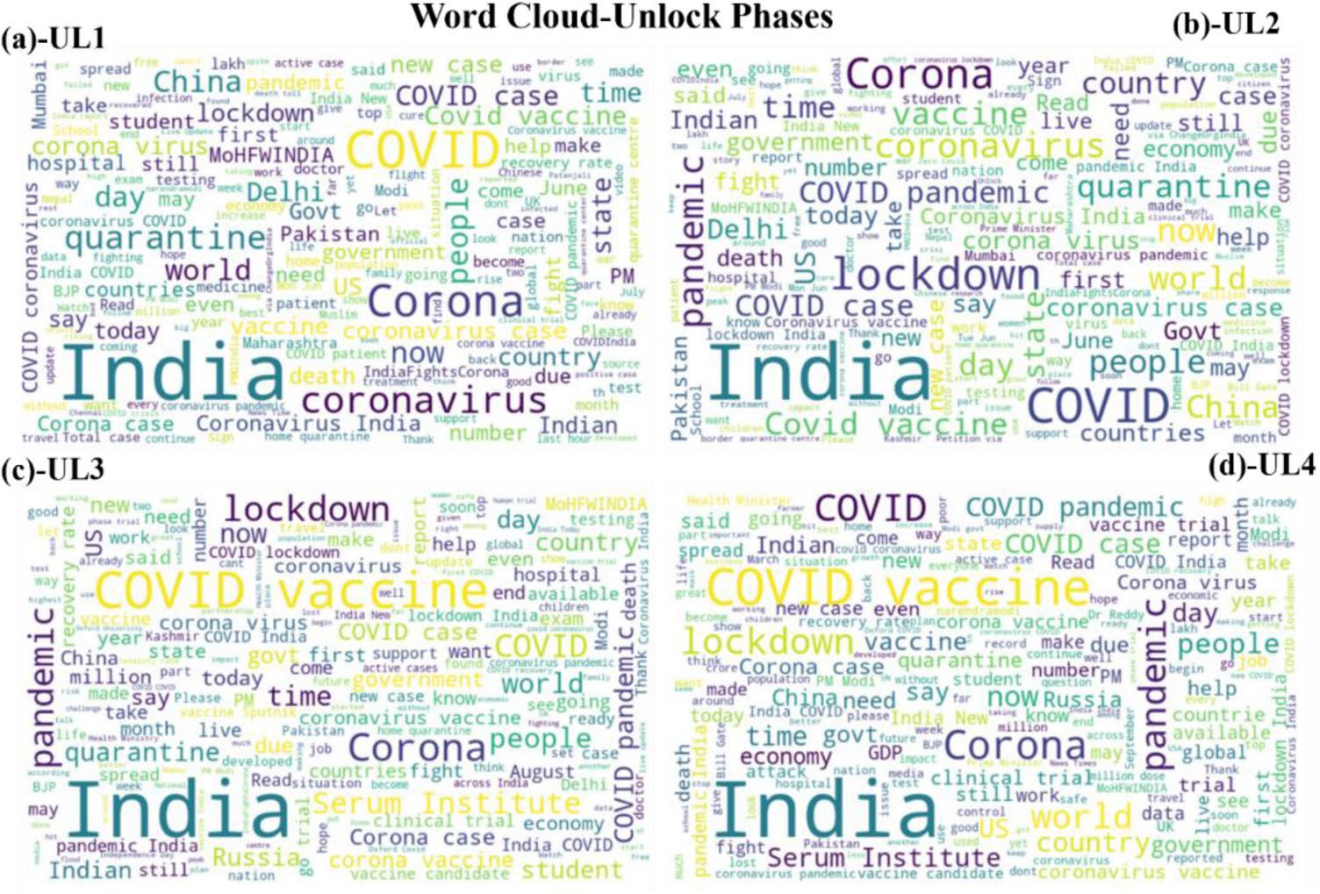
Word cloud during lockdown phases (**a**) lockdown 1 (L1) (**b**) lockdown2 (L2) (**c**) lockdown 3 (L3) (**d**) lockdown4 (L4).

**Figure 3:**
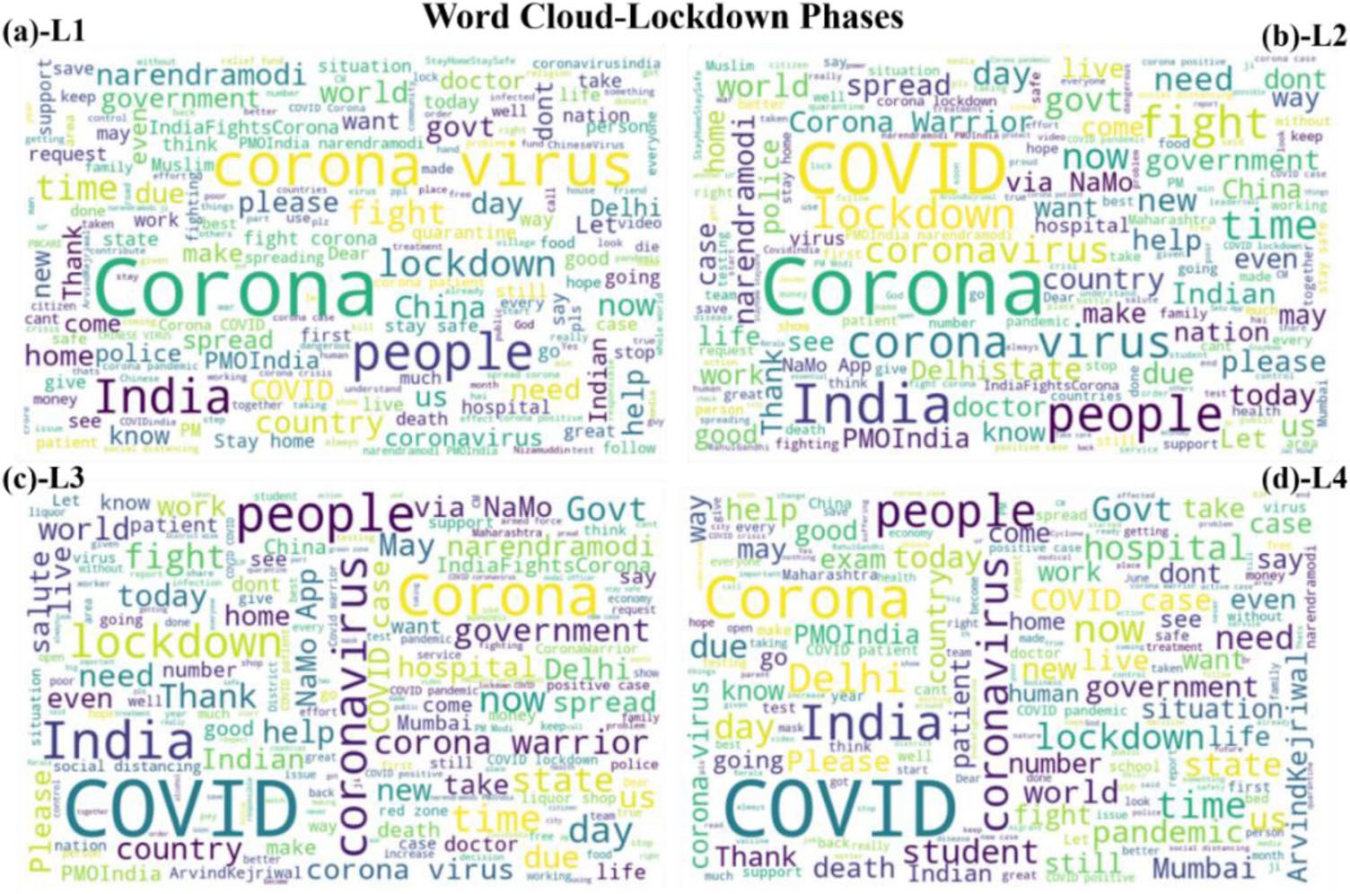
Word cloud during unlock phases (**a**) unlock 1 (UL1) (**b**) unlock (UL2) (**c**) unlock 3 (UL3) (**d**) unlock (UL4).

Before applying the trained model on the datasets it was essential to understand the trend of the tweets during the different lockdown and unlock phases. Wordcloud of the most occurring words were calculated to quantify and understand the trend. It can be observed that most used words kept changing during the periods. At the same time, words related to coronavirus, lockdown, and government were the most tweeted during L1 to L2 (see Fig. 2). During the lockdown period people were suffering from resource-related difficulties in addition to adjusting and fighting the coronavirus. On the other hand government and agencies were constantly spreading the information related to safety, procedures and protocols. Thus, the tweets and most prominent words reflect the activities and situation during the lockdown. During unlock phases the focus was on opening the country by restarting the closed activities. Moreover, the buzz was about the many vaccines in development across the world. Hence, ‘vaccine’, and related topics such as ‘Serum Institute’, ‘Russia’ were prominent words apart from the virus-related words (see Fig. 3). During the unlock period a hype around restarting the economy and failing Gross Domestic Production (GDP) was also a topic of discussion of the citizens.

## Model accuracy evaluation

The models were implemented using opensource Keras Python Library with TensorFlow backend. The training process was implemented on a personal computer with a i7-8750H CPU, 16 GB of RAM, a × 64-based processor, and a 4 GB Nvidia graphics card. The parameters were selected after various trials for better accuracy. The model was trained for 11 epochs for batch size 512. ‘Adam’ algorithm was used to update network weights in the training process to optimize the model. The prediction accuracy of the model was carried out to analyze their performance. The accuracy metric is the ratio of correct predictions to the models total prediction (4).

4 $${\text{Accuracy}} = { }\frac{{{\text{Correct}}\;{\text{Predictions}}}}{{{\text{Total}}\;{\text{ Predictions}}}}$$

**Figure 4:**
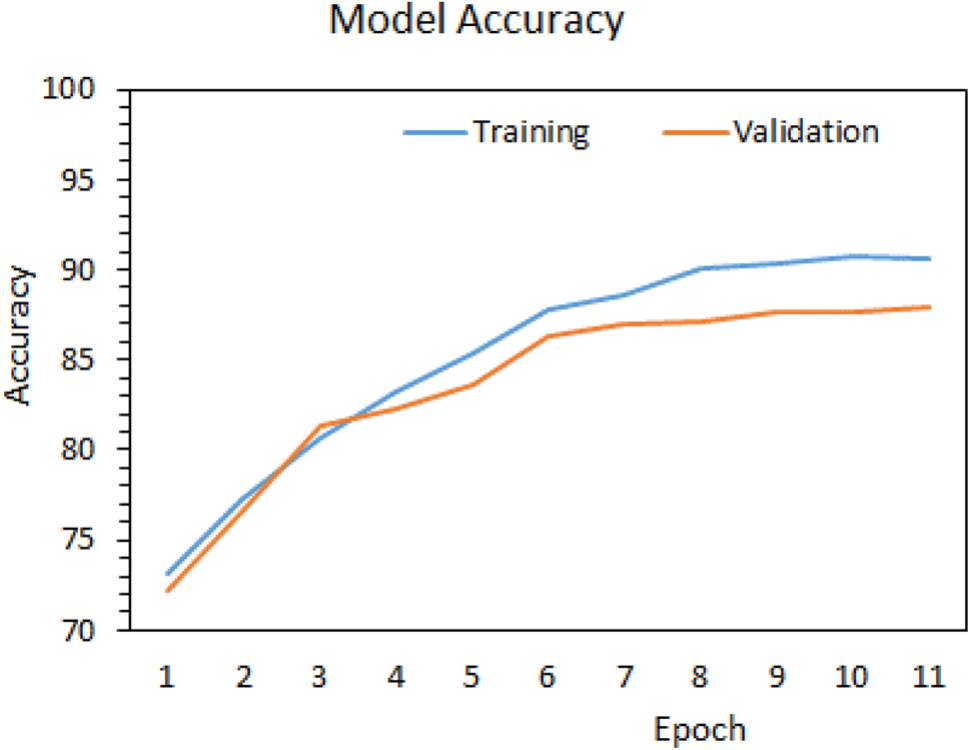
Model accuracy graph on training and validation sets.

Figure 4 shows the accuracy graph of model for training and validation datasets. It can be observed that model performed very well. When applied to the test dataset the model reported accuracy of around 90%. To emphasize the importance of the proposed architecture the network was changed in the following ways: (a) CNN layer was put before BiLSTM, (b) only considered individual CNN model, (c) only considered individual BiLSTM model. For unbiased comparison, the execution parameters were kept the same as the proposed model (see Table 4). It was observed that CNN and BiLSTM alone (case b and c) gave the least accurate result. Table 5 details the attained accuracy on the test datasets by the models. Although our proposed model was more accurate than the rest, it took significantly more time to train than the case (a) model. Hence, a trade-off of execution time exists, which can be addressed based on the requirements.

**Table 5:** Accuracy achieved by the models on test dataset.

Model	Test accuracy
***Proposed***: BiLSTM + CNN	89.68%
CNN + BiLSTM	87.38%
LSTM	86.65%
CNN	85.20%

## Sentiment classification results and their GIS based analysis

**Figure 5:**
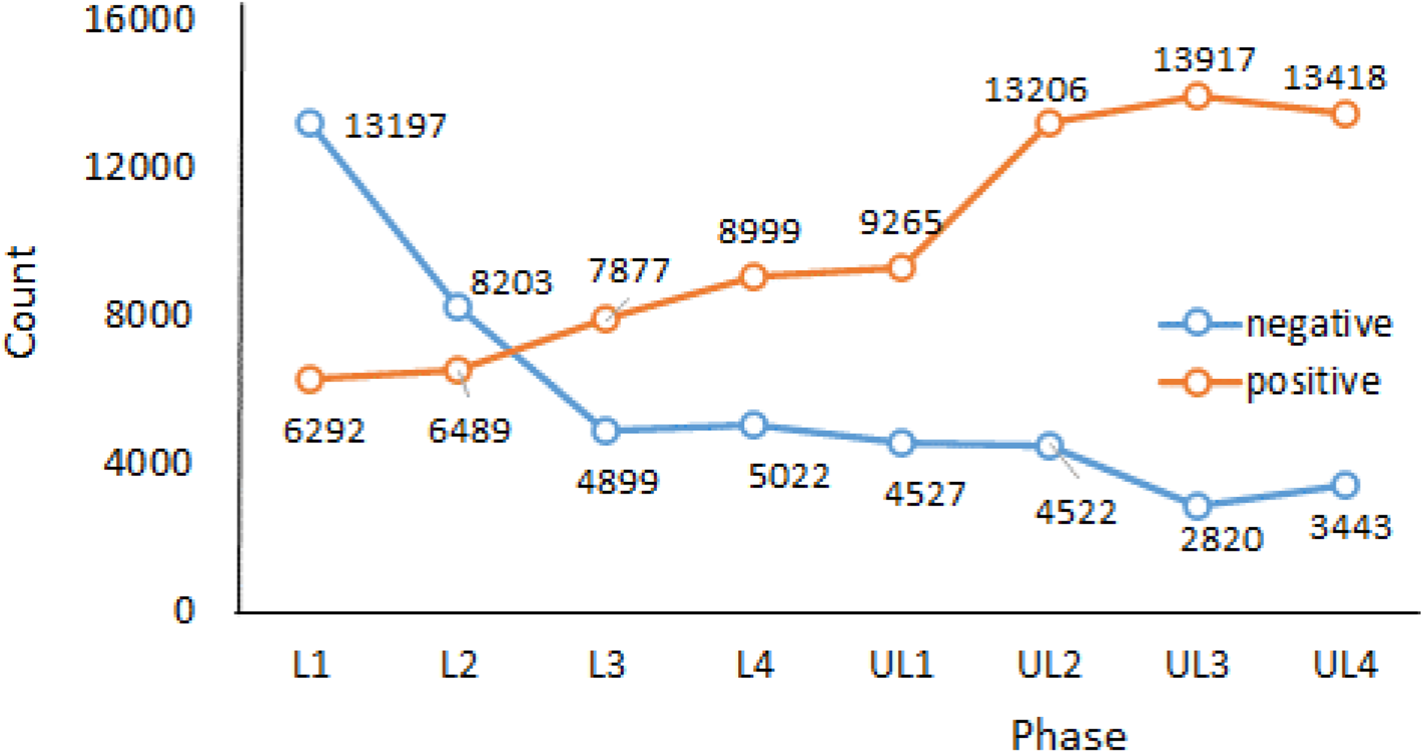
Predicted count of tweet sentiments during lockdown and unlock phases.

**Figure 6:**
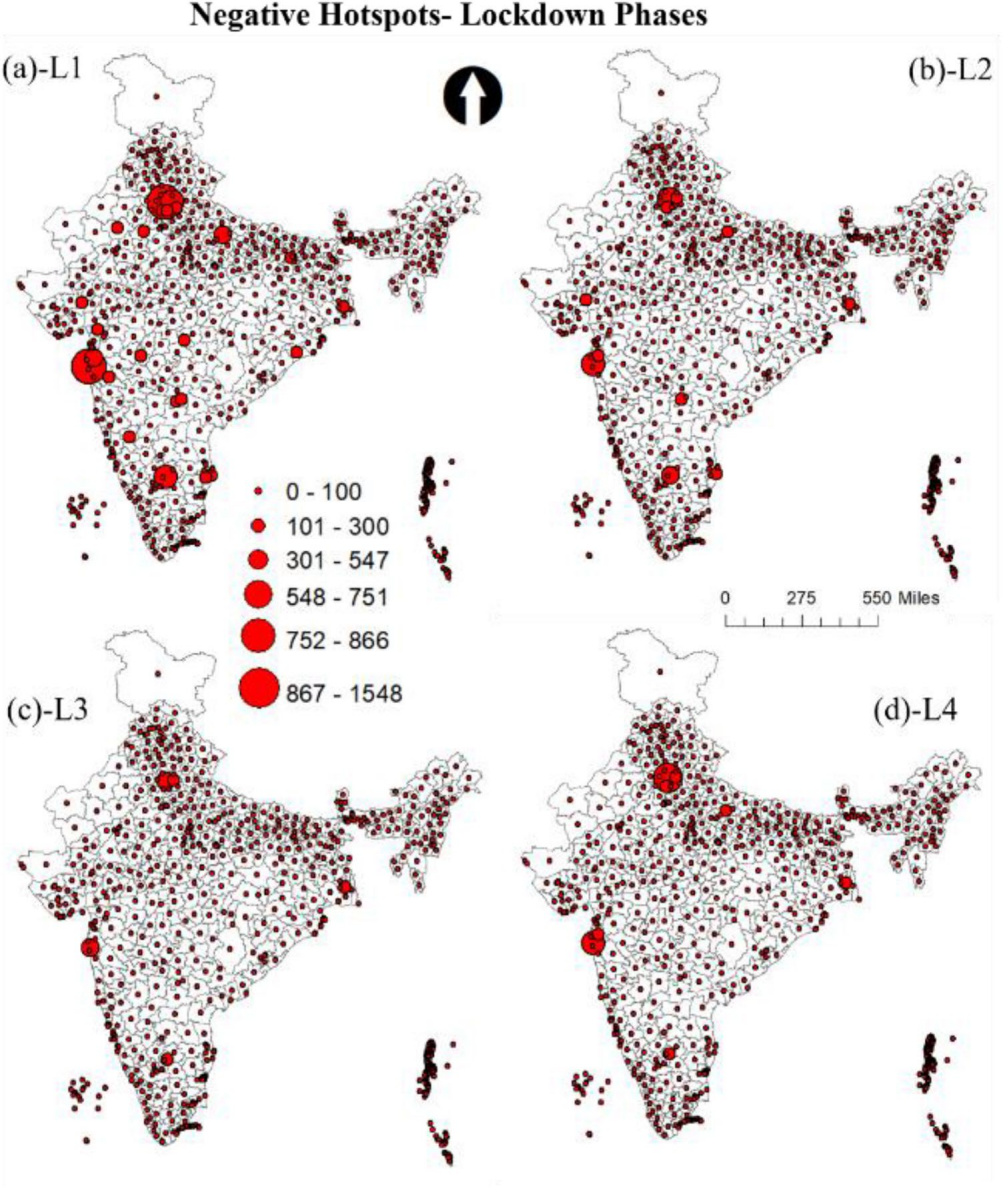
Negative sentiment count hotspots during lockdown phases (**a**) lockdown 1 (L1) (**b**) lockdown2 (L2) (**c**) lockdown 3 (L3) (**d**) lockdown4 (L4).

**Figure 7:**
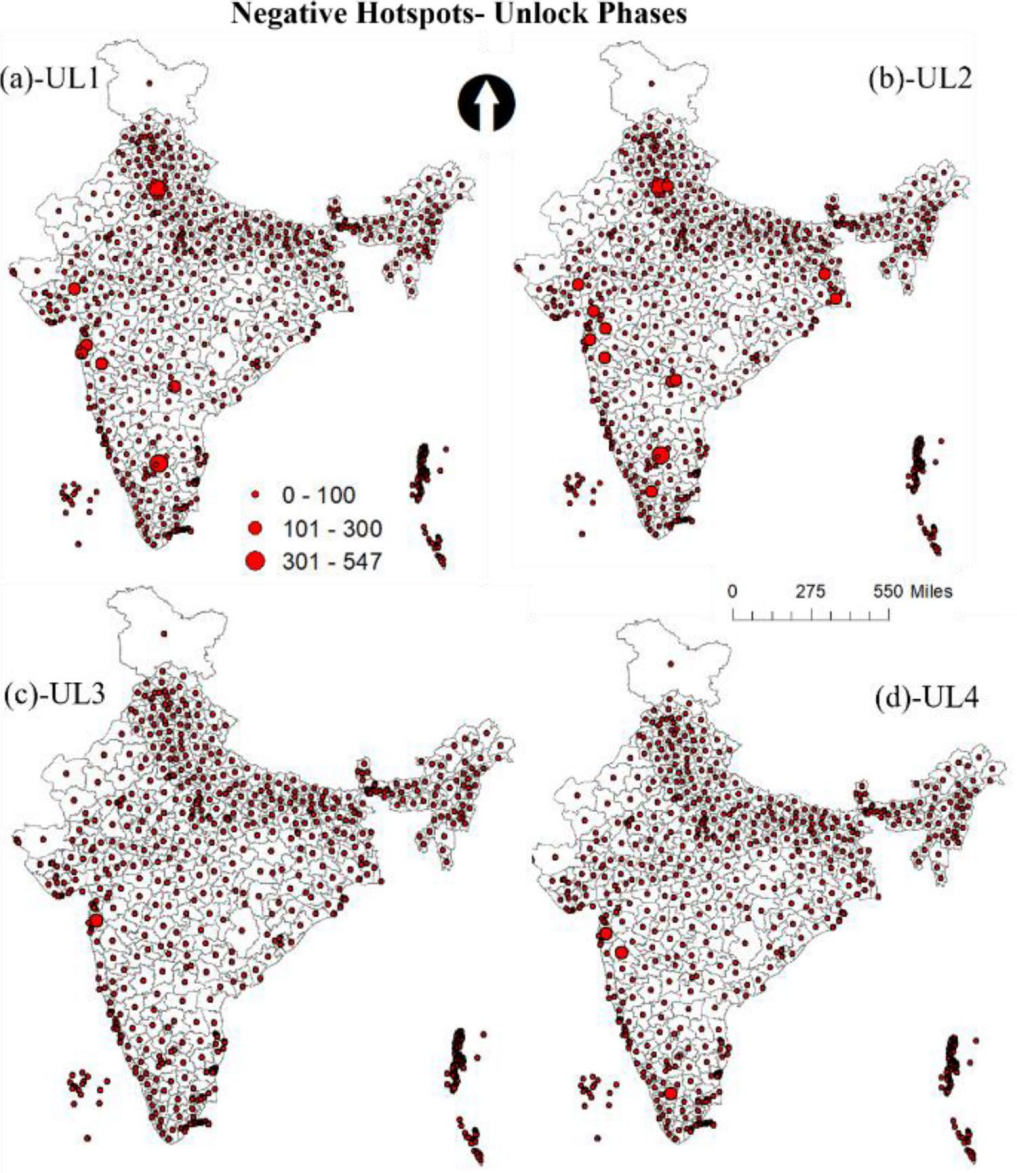
Negative sentiment count hotspots and during unlock phases (**a**) unlock 1 (UL1) (**b**) unlock (UL2) (**c**) unlock 3 (UL3) (**d**) unlock (UL4).

Figure 5 shows the predicted count graph of the tweet sentiments during lockdown and unlock phases. From the graph, there is a clear trend in the reduction of negative tweets and increase of positive tweets during lockdown to unlock phases. To better understand the spatial patterns and the trends of the sentiments, the study utilizes the advantage of geotagged tweets. GIS was used to spatially join the predicted sentiments to the Indian district's spatial data. Aggregation operation was then performed to deduce the total count of each sentiment in every district. The final aggregated outcome was then used to develop graduated symbolic maps based on count for the data. FigureS 6 and 7 show the resultant negative sentiment hot spot maps for lockdown and unlock phases, respectively. Substantial high variations in the location clusters and the sentiment count can be observed during lockdown and unlock phases. The unlock phases accounted for very fewer negative tweets compared to the lockdown phases, as the unlock phases were the step taken for bringing normalcy to daily life of the citizens by allowing many activities, including the opening of travel models such as railways, office with certain percentage of employees, and food joints.

The metropolitan cities such as Delhi, Mumbai, and Bangalore witnessed most negative sentiment tweets. This was reasonable as the urban agglomerations were the hotspots of COVID-19 cases, and citizens faced severe resource crises during the lockdown. A significant difference in the total count of tweets during the lockdown phases (L1- L4) can also be noticed. The highest number of negative tweets were done during L1, which is primarily because it was a first lockdown experience. The same period witnessed the massive out-migration of marginalized section of the society from metro cities, in the lack of food, resources and fear of the virus spread. Further, a large section of society was in favour of lockdown much earlier. The difficulties related to daily needs, travel and rising cases drew severe reactions from the citizen on social media. On the other hand, the negative tweets during lockdown were least during L3, which might be because some ease on lockdown activities were allowed (see Table 1) and citizens were accustomed to lifestyle amidst the lockdown. Besides, government during the L1 and L2 laid a plan according to which various agencies had started the production of masks, sanitizer, and facilities such as COVID-19 specialty hospitals were set up. Moreover, based on the media reports people had a feeling of end of lockdown period with decrease in cases, which also contributed to less negative sentiments during the last lockdown phases.

**Figure 8:**
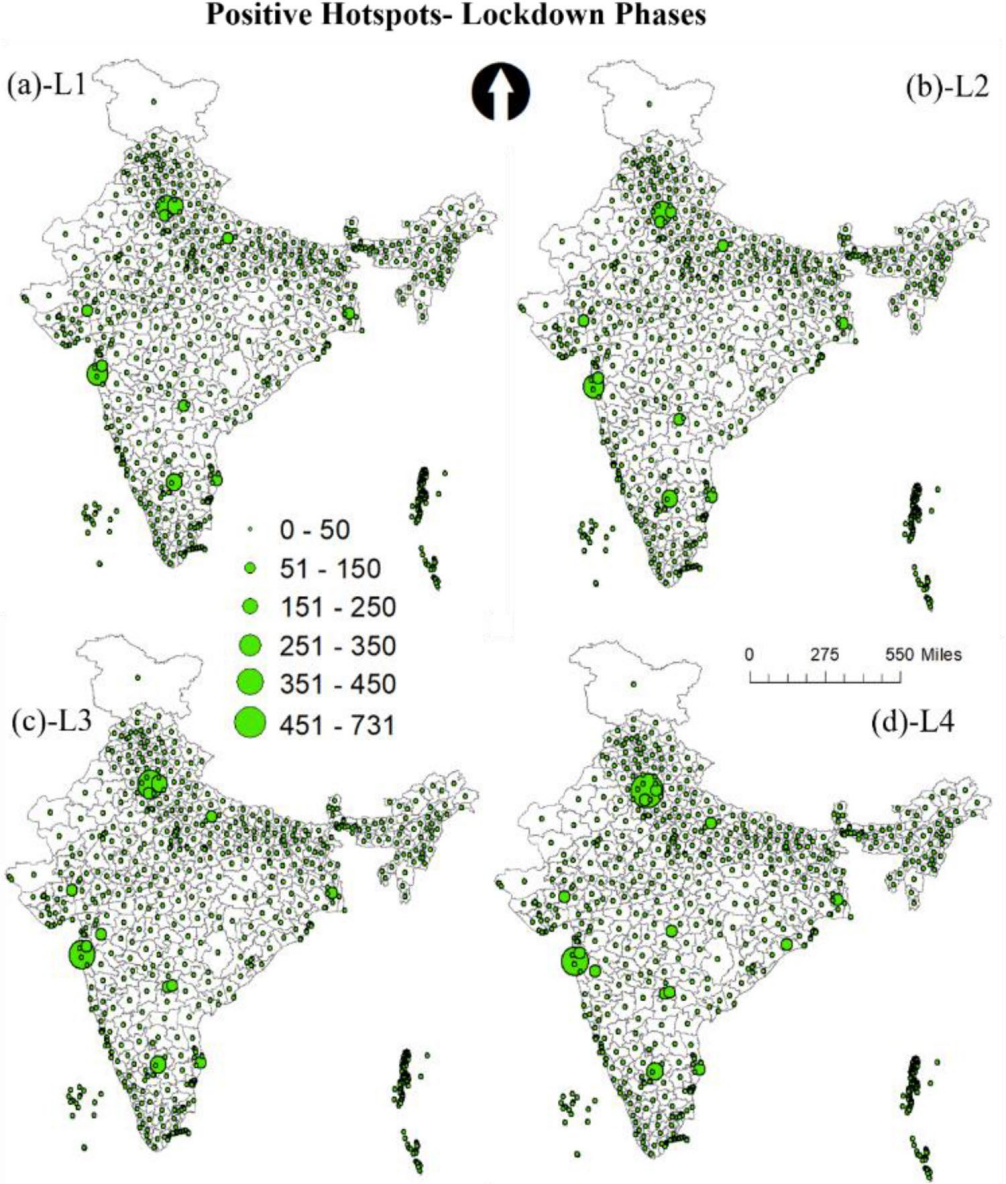
Positive sentiment count hotspots during lockdown phases (**a**) lockdown 1 (L1) (**b**) lockdown2 (L2) (**c**) lockdown 3 (L3) (**d**) lockdown4 (L4).

Although the virus and lockdown had led to negativity, sadness, fear, and disgust, there were still many events and instances of positivity. This is evident in Fig. 8, which shows the positive sentiments hotspots and district-based count during the lockdown phases. The positivity among the citizen was mainly due to efforts by agencies and organizations that implemented the plans so that basic essentials were arranged during lockdown. The awareness among the citizen regarding the measures required by them to overcome the challenges faced during the pandemic in longer run was also a factor. The events such as clean air and rivers due to least human activity also drew positive reactions. However, the positive tweets were less than the negative during the lockdown phases, which is reasonable since the lockdown had posed huge challenges in front of citizens and agencies to make it successful. It can be observed that most positive tweets during lockdown were done during the L4, which was announced as the last lockdown primarily due to the concerns over slowing GDP. The wordcloud (see Fig. 3) also shows GDP as one of the frequently tweeted words during the last phase.

**Figure 9:**
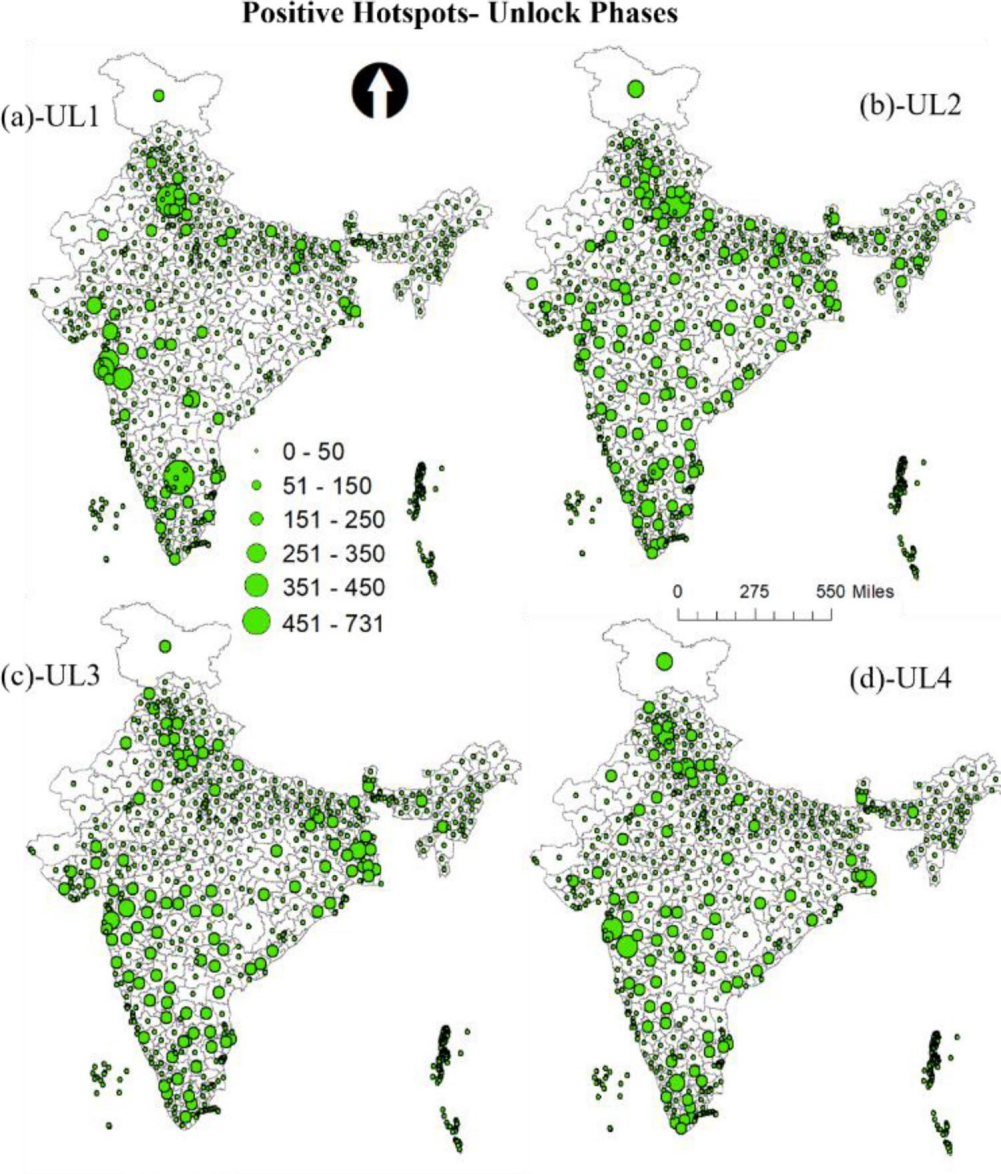
Positive sentiment count hotspots during unlock phases (**a**) unlock 1 (UL1) (**b**) unlock (UL2) (**c**) unlock 3 (UL3) (**d**) unlock (UL4).

On the other hand, the positive sentiment was very high throughout the country during unlock period, which was expected as the unlock phases were step towards bringing normalcy in the daily activities. Figure 8 illustrates the high count of positive sentiments across the country. One interesting observation is the spread of positive hotspots during U3 and U4, in which the tweets were considerably higher compared to any phase for many parts of the country (see Fig. 9). This was primarily because during the period not only many activities were allowed, industries, office, schools opened to boost the economy which had taken a backseat during the lockdown.

## Discussion and utility of the research

From the outcome of the model, it was observed that hotspots of negative and positive sentiments were mostly from the regions where cases were very high, specifically in the metropolitan regions. Moreover, with every phase towards normalcy the geotagged negative tweets reduced and positive tweets increased. In most cases, people seem to be divided over the trending topics as it was observed that the same regions witnessed both negative and positive hotspots. The positive sentiment hotspots were more in the unlock period compared to the lockdown; this shows that with time people were happy with the proceedings. Besides, the positive sentiments during lockdown point to its success by controlling the spread reasonably. Moreover, the negative sentiment count decreased significantly during the unlock period. A large number of tweets were used in the analysis; still, it will be interesting to see how the hotspot pattern will look if all the tweets would have been geotagged. The topics that were tweeted kept changing during the phases. Using the wordcloud, topics related to COVID-19, safety, vaccine, help, etc., were the most frequent word during all the phases. However, words such as economy GDP, show that with time people started talking about related outcomes of the lockdown phases such as decreasing GDP and talks about the economy in the bad stage.

The model’s outcome demonstrates that hybrid deep learning models can be a very good tool for sentiment prediction. Moreover, a novelty of the paper lies in the fact that the model captures the context of the text, which is generally new as the coronavirus is a novel virus, and so were the tweeted words. The tweets and their context required an approach that can capture the semantics and the local relationships. Concerning sentiment analysis of COVID-19 tweets, some papers have applied the unsupervised modeling approach to predict the sentiment, but this paper's supervised approach makes it foremost in the area, especially for India, a subcontinental region.

**Figure 10:**
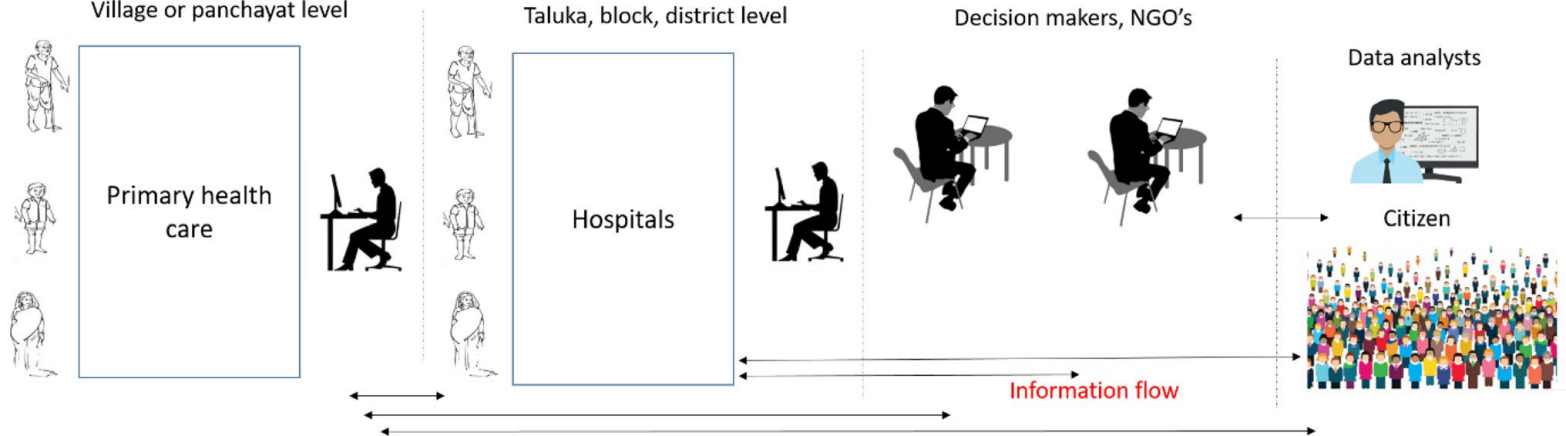
A conceptual framework that uses social media analytics for social benefits during pandemic like COVID-19.

It is still not clear that topics related to COVID-19 pandemic will be a focal point of discussion on social media. It seems it will stay with us until a significant population across the globe are vaccinated. For a resource constraint country like India, every piece of information must be used for public benefits. Social media analytics is one such medium that can help in handling many situations and requirements of citizens until humans overcomes this crisis. With a focus on the resources, the specific keywords such as “less beds” “medicines”, “doctor”, the tweets can further be used in identifying resource details based on the sentiments. Suppose the study is combined with an architecture in which the information extracted from a user’s tweets and decision-makers is used. In that case, such a system can be a potent approach to handle not only COVID-19 but any bio and natural disasters. Figure 10 shows such a proposed conceptual decision-making system in which verified tweet handles can be used to tweet the hospital resource details to stakeholders such as NGOs, citizens, data scientists, and agencies. The prediction and language processing models can extract valuable information in informed decision-making.

## Conclusion

The study addresses the gap in analysing the spatiotemporal pattern of sentiments of COVID-19 related geotagged tweets for India done during lockdown and unlock phases. The sentiments were predicted by the proposed hybrid deep learning model. The model integrates bidirectional long term short memory (BiLSTM) and convolutional neural network (CNN) model classes to leverage their benefits. The model trained over Sentiment140 dataset was applied to predict the sentiments.

The results show significant variations in the sentiment location hotspots and their count. The metropolitan cities that witnessed most cases were found to be the prime hotspots of negative sentiments. Many areas, predominantly metropolitan cities, also witnessed high positive tweets during unlock periods. Besides, it was observed that the negative tweets decreased and positive tweets increased during the gradual lockdown and unlock phases. The paper outcomes as a decision support system can be used in better resource management (e.g., vaccination, emergency response, infrastructure management etc.) and policy formulation during the ongoing COVID-19 crisis. Moreover, government should conduct awareness programs for citizens to encourage them regarding geotagging their tweets, as it helps in analyzing the location based requirements.

India has many official languages; however, the study only considered tweets done in the English language. This might have lead to some loss of information. In future work, the proposed study can be extended to analyse multilingual texts. Current study considers two tweet classes i.e., negative and positive. However, once the annotated data related to COVID-19 having neutral sentiments is accessible to the research community the current work can be extended to consider the same. Moreover, it will be interesting to observe the sentiment pattern of the tweets done after the discussed lockdown and unlock phases for further analysis.

## Data Availability

The datasets and codes generated during and/or analysed during the current study are available from the corresponding author on reasonable request.
